# Risk prediction models for feeding intolerance in patients with enteral nutrition: a systematic review and meta-analysis

**DOI:** 10.3389/fnut.2024.1522911

**Published:** 2025-01-14

**Authors:** Huijiao Chen, Jin Han, Jing Li, Jianhua Xiong, Dong Wang, Mingming Han, Yuehao Shen, Wenli Lu

**Affiliations:** ^1^Department of Neurosurgery, Tianjin Medical University General Hospital, Tianjin, China; ^2^Department of Critical Care Medicine, Tianjin Medical University General Hospital, Tianjin, China; ^3^Department of Epidemiology and Health Statistics, Tianjin Medical University, Tianjin, China

**Keywords:** enteral nutrition, feeding intolerance, risk prediction model, systematic review, meta-analysis

## Abstract

**Background:**

Although more risk prediction models are available for feeding intolerance in enteral-nourishment patients, it is still unclear how well these models will work in clinical settings. Future research faces challenges in validating model accuracy across populations, enhancing interpretability for clinical use, and overcoming dataset limitations.

**Objective:**

To thoroughly examine studies that have been published on feeding intolerance risk prediction models for enteral nutrition patients.

**Design:**

Conducted a systematic review and meta-analysis of observational studies.

**Methods:**

A comprehensive search of the literature was conducted using a range of databases, including China National Knowledge Infrastructure (CNKI), Wanfang Database, China Science and Technology Journal Database (VIP), SinoMed, PubMed, Web of Science, The Cochrane Library, Cumulative Index to Nursing and Allied Health Literature (CINAHL) and Embase. The search scope was confined to articles within the database from its inception until August 12th, 2024. The data from the selected studies should be extracted, including study design, subjects, duration of follow-up, data sources, outcome measures, sample size, handling of missing data, continuous variable handling methods, variable selection, final predictors, model development and performance, and form of model presentation. The applicability and bias risk were evaluated using the Prediction Model Risk of Bias Assessment Tool (PROBAST) checklist.

**Results:**

A total of 1,472 studies were retrieved. Following the selection criteria, 18 prediction models sourced from 14 studies were incorporated into this review. In the field of model construction, only one study employed the use of multiple machine-learning techniques for the development of a model. In contrast, the remaining studies used logistic regression to construct FI risk prediction models. The incidence of FI in enteral nutrition was 32.4–63.1%. The top five predictors included in the model were APACHE II, age, albumin levels, intra-abdominal pressure, and mechanical ventilation. The reported AUC, or area under the curve, exhibited a range of values between 0.70 and 0.921. All studies were identified as having a high risk of bias, primarily due to the use of inappropriate data sources and inadequate reporting within the analysis domain.

**Conclusion:**

Although the included studies reported a certain degree of discriminatory power in their predictive models to identify feeding intolerance in patients undergoing enteral nutrition, the PROBAST assessment tool deemed all the included studies to carry a significant risk of bias. Future research should emphasize the development of innovative predictive models. These endeavors should incorporate more extensive and diverse sample sizes, adhere to stringent methodological designs, and undergo rigorous multicenter external validation to ensure robustness and generalizability.

**Systematic review registration:**

Identifier CRD42024585099, https://www.crd.york.ac.uk/prospero/display_record.php?RecordID=585099.

## 1 Introduction

Enteral nutrition feeding intolerance (FI) refers to patients experiencing gastrointestinal adverse reactions during enteral nutrition, such as vomiting, high gastric residual volume, diarrhea, and gastrointestinal bleeding. Enteral nutrition cannot achieve its nutritional goals after 72 h, or it needs to be terminated for clinical reasons (1, 2). FI is an external reflection of the gastrointestinal dysfunction (3). According to literature reports, the incidence of FI in patients with enteral nutrition is 2 to 75% (4), which mainly occurs within 1 to 3 days after the start of enteral nutrition and lasts for about a week (5). FI can interrupt enteral nutrition support (6), leading to insufficient nutrient intake and substandard feeding rates, resulting in malnutrition, low immunity, insufficient energy metabolism in patients, and an increased risk of aspiration pneumonia. Continued uncontrolled FI can lead to intestinal mucosal barrier damage, intestinal flora displacement, and bloodstream infection in patients with enteral nutrition (7). It directly affects the clinical outcome of patients and increases the difficulty and workload of nursing. Studies have indicated that FI represents a notable risk factor for adverse prognosis and heightened mortality rates within a three among patients receiving enteral nutrition feeding (8, 9). Therefore, early identification of high-risk patients with FI and early intervention against risk factors are essential to prevent FI in patients receiving enteral nutrition.

Predictive models employ multifactor models to ascertain the likelihood of developing a disease or experiencing a future outcome. A risk prediction model represents the initial stage in the prevention of FI. The precision of the forecasting outcomes subsequently impacts the choice of suitable preventative actions and their effectiveness. A risk prediction model for enteral feeding intolerance is a quantitative instrument that utilizes various predictors to assess the likelihood or danger of developing enteral feeding intolerance (10). The predictors above may comprise general indicators about the subject’s current and historical medical history and those derived from laboratory tests. Establishing the FI risk prediction model can change the perspective of nursing staff from treatment and care after FI occurrence to purposeful and focused assessment before FI occurrence, giving preventive measures in advance and risk factors and protective factors determined by the risk prediction model. It is conducive to improving the accuracy and comprehensiveness of FI assessment by medical staff, thereby guiding the clinical development of personalized nursing plans. Moreover, it can facilitate the implementation of targeted preventive measures to enhance patients’ tolerance to enteral nutrition, thereby improving their clinical outcomes. Therefore, developing a dedicated risk prediction model for feeding intolerance in patients receiving enteral nutrition is crucial. Despite the increasing number of such risk prediction models, there has been no comprehensive assessment of their quality and applicability. Specifically, the existing models suffer from several limitations, including insufficient sample sizes, inappropriate selection of predictors, and lack of validation in diverse patient populations. These limitations hinder their effectiveness and reliability in clinical practice. There is no unified and recognized model for predicting FI in patients with enteral nutrition, which has certain restrictions, and the clinical management of FI in patients with enteral nutrition still faces great challenges (11). The objective of this study was to conduct a systematic review and evaluation of the extant models for predicting FI in patients with enteral nutrition. The results will constitute a valuable source of reference for the practice of medicine and the planning of future research projects.

## 2 Methods

The protocol for the study has been officially documented on PROSPERO, bearing the registration number CRD42024585099.

### 2.1 Search strategy

Given the substantial population base and the widespread linguistic use, we embarked on an exhaustive search encompassing both Chinese and English databases. The following databases were consulted in the course of the present study: China National Knowledge Infrastructure (CNKI), Wanfang Database, China Science and Technology Journal Database (VIP), SinoMed, PubMed, Web of Science, The Cochrane Library, Cumulative Index to Nursing and Allied Health Literature (CINAHL) and Embase. The search terms were inputted into the aforementioned databases between their inception and 12 August 2024, using the following keywords: “Enteral nutrition,” “Feeding intolerance,” “Feeding tolerance,” “Risk prediction model,” “Risk factor,” “Predictor” “Model” “Risk Score.” To illustrate, a detailed search was conducted using PubMed, which entailed the following:

#1 “Enteral nutrition” [MeSH Terms] OR “Feeding intolerance” [Title/Abstract] OR “Feeding tolerance” [Title/Abstract].

#2 “Risk prediction model” [Title/Abstract] OR “Risk factor” [Title/Abstract] OR “Predictor” [Title/Abstract] OR “Model” [Title/Abstract] OR “Risk score” [Title/Abstract].

#1 AND #2.

The Supplementary material provides an overview of the comprehensive search methodologies utilized for various databases. Additionally, pertinent studies were further identified by examining the bibliographies of the retrieved research papers and review articles.

The systematic review adhered to the PICOTS framework, as recommended by the CHARMS checklist, for the purposes of rigorously evaluating and extracting data from studies on prediction modeling within the context of systematic reviews (12). The system aids in clearly defining the objective of the review, outlining the methodology of the search strategy, and establishing the criteria for including and excluding studies (13). Below, we describe the essential aspects of our systematic review:

P (Population): Patients ≥18 years old with initiation of enteral nutrition within 48 h of admission.
I (Intervention model): Risk prediction models for feeding intolerance, specifically tailored for patients undergoing enteral nutrition, have been formulated and later published. These models incorporate at least two predictors (i.e., predictors ≥2).
C (Comparator): No competing model.
O (Outcome): The outcome of interest was specifically FI rather than any of its subgroups.
T (Timing): The prediction of the outcome was derived from an assessment encompassing admission details, clinical scoring results, and lab test indicators.
S (Setting): The objective of the FI risk assessment model lies in tailoring forecasts specifically for patients undergoing enteral nutrition, thereby facilitating the implementation of preventive strategies to mitigate potential adverse effects.

### 2.2 Inclusion and exclusion criteria

The inclusion criteria for the studies encompassed: (1) Studies that include patients aged 18 and above who commenced enteral nutrition within the initial 48 h following admission; (2) Adoption of an observational research framework; (3) Development and exhibition of a forecasting model; and (4) a primary outcome focus on feeding intolerance (FI).

Conversely, the exclusion criteria were as follows: (1) Research endeavors that failed to establish a predictive model; (2) Studies reporting outcomes exclusively about FI (feeding intolerance) subgroups; (3) Publications that were not authored in English or Chinese; and (4) Instances where, despite attempts to reach the authors via email, access to the complete text remained unattainable.

### 2.3 Study selection and screening

The screening studies involved two authors, HJ and CHJ, working independently. Initially, any duplicate studies were removed. Following this, the eligibility of the remaining studies was evaluated by examining their titles and abstracts. After applying the inclusion and exclusion criteria, a comprehensive review of their full texts was conducted. Furthermore, a thorough examination of the reference lists of all eligible studies was conducted to identify any potentially relevant studies that might have been missed. In cases where disagreements arose during the study selection process, the third author, SYH, was involved in the discussion until a unified decision was reached. To quantify interrater agreement, we calculated an unweighted Cohen’s kappa (*κ*) value (14).

### 2.4 Data extraction

Two independent reviewers screened and assessed the suitability of full-text articles, resolving disagreements through discussion or with a third reviewer’s input. To ensure the accuracy of data extraction, we also used the following statistical methods to assess interrater agreement: unweighted Cohen’s kappa (*κ*) values were calculated for binary or nominal variables; For continuous variables, two-way intraclass correlation coefficients (ICCs) were calculated (15).

The information extracted from the chosen studies was categorized into two groups: (1) Basic details included the author, publication year, research design, participant attributes, data origin, and sample size. (2) Specifics related to the prediction model included details on variable selection methods, model development strategies, validation types, performance measures, strategies for addressing missing data, handling of continuous variables, the final predictors in the model, and the presentation format of the model. A reviewer was responsible for extracting the information, and another independently confirmed its precision and uniformity.

### 2.5 Quality assessment

For assessing the quality and potential bias risks in the studies included, we adopted two evaluation instruments: the Grading of Recommendations, Assessment, Development, and Evaluation (GRADE) system (16) and the accessible version of the Prediction Model Risk of Bias Assessment Tool (PROBAST) (17). The objective was to evaluate the likelihood of bias in the studies included and offer insights into their overall quality.

The GRADE framework categorizes research findings and assesses the quality of evidence, considering six key factors: study design, risk of bias, inconsistency, indirectness, imprecision, and supplementary considerations like publication bias. This approach is specifically designed to evaluate the quality of evidence in systematic reviews. The PROBAST checklist was utilized to assess the potential bias and the applicability concerns of the studies included. Two authors, namely HJ and CHJ, independently conducted evaluations of bias and applicability issues—the PROBAST checklist aids in critically reviewing studies on developing, validating, or refining individualized prediction models. The tool consists of 20 key questions categorized into four areas: participants, predictors, outcomes, and analytical approaches. Responses to each inquiry can be provided as “yes,” “probably yes,” “no,” “probably no,” or an indication of “no information.” If any inquiry within a particular area receives a response of “no” or “probably no,” that area is flagged as posing a heightened risk of bias. Only when all areas are deemed to have a low risk of bias can the overall assessment be considered to present a low risk of bias?

### 2.6 Data synthesis and statistical analysis

A qualitative systematic review methodology was utilized to categorize and synthesize the core attributes of the incorporated studies, the construction and validation procedures of the model, and its operational performance. A thorough meta-analysis was conducted on the area under the receiver operating characteristic curve (AUC) values derived from the validated models, employing the Stata software (version 18.0; Stata Corporation, College Station, Texas, USA). Furthermore, a meta-analysis was carried out on the five most significant predictors identified from the model, utilizing the aforementioned Stata software. Both the *I^2^* statistic and the Cochrane Q-test were used to evaluate the extent of heterogeneity among the studies. The *I^2^* index measures heterogeneity, with 25, 50, and 75% indicating low, moderate, and high levels, respectively. If *P* is more significant than 0.1 and *I^2^* is equal to or less than 50%, it can be concluded that there is no statistical heterogeneity among the studies in question. Consequently, the fixed effect model was employed for the subsequent analysis. If *P* is less than 0.1 and *I^2^* is greater than 50%, it can be concluded that there is a high degree of heterogeneity among the studies. In such instances, the random-effects model is employed for the analysis (18). A sensitivity analysis was conducted by sequentially excluding individual articles to observe whether there was a significant change in heterogeneity after excluding articles and to observe the change in the pooled effect size. Suppose the heterogeneity changed significantly after excluding articles one by one. In that case, the article may be the source of heterogeneity, and the reason for it to be the source of heterogeneity was analyzed. To detect potential publication bias, Egger’s regression test was employed, yielding a *p*-value exceeding 0.05, which indicated a minimal likelihood of such bias existing (19).

## 3 Results

### 3.1 Study selection

**Figure 1:**
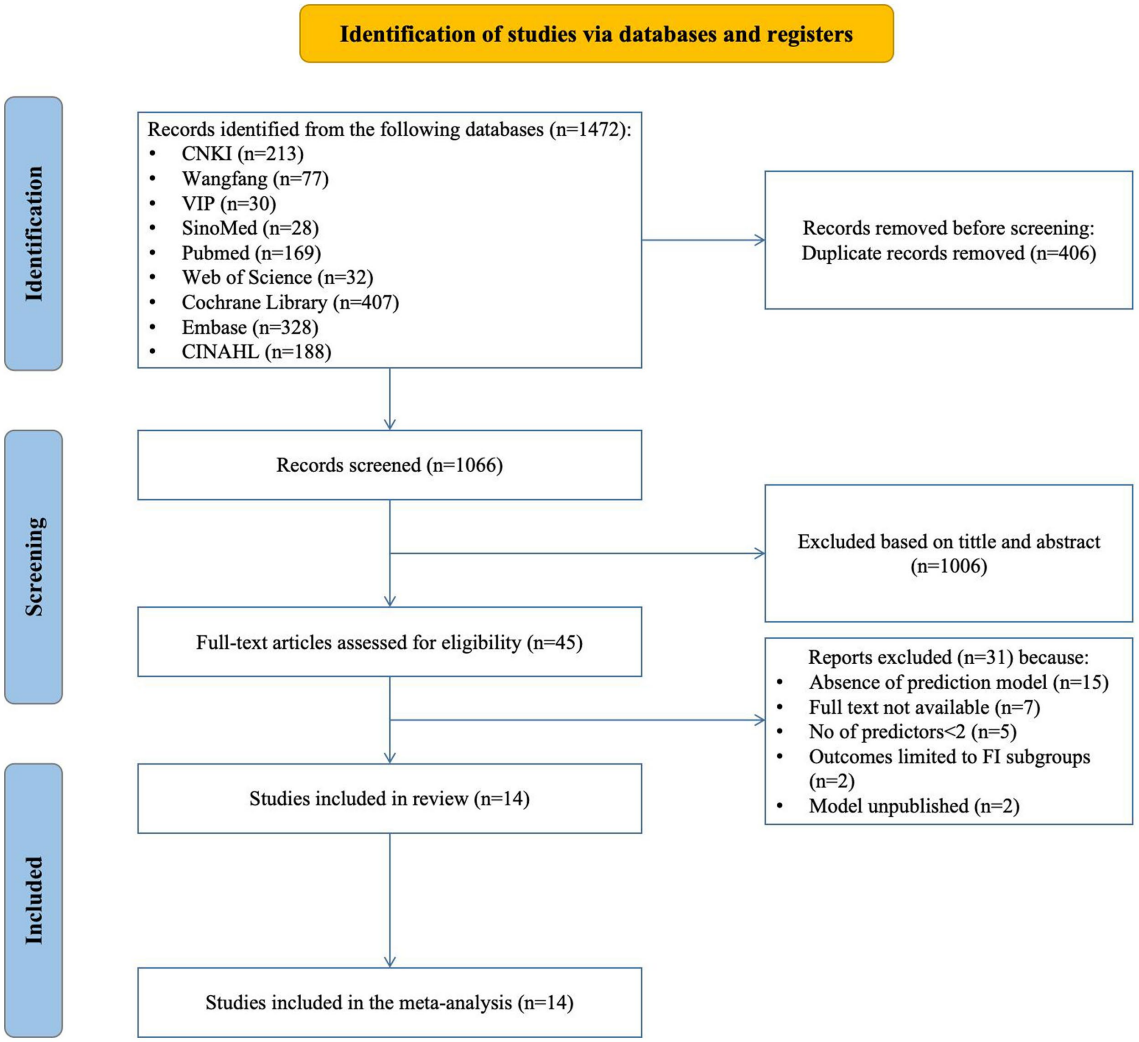
Preferred reporting items for systematic REVIEWS and meta-analyses (PRISMA) flowchart of literature search and selection.

Figure 1 displays the Preferred Reporting Items for Systematic Reviews and Meta-Analyses (PRISMA) 2020 flow diagram, which illustrates the extensive search methodology and the outcomes obtained during the process.

The search returned 1,472 articles; after removing 406 duplicates, 1,066 were screened for eligibility. This screening led to the selection of 45 articles for further assessment. During this subsequent evaluation, we excluded 15 studies due to their lack of prediction model development or exclusive focus on risk factors. Additionally, 7 studies were inaccessible in full-text format, 5 studies possessed fewer than two predictors, outcomes in 2 studies were confined to particular subgroups, and another 2 studies should have published their predictive models. Consequently, 14 studies, encompassing 18 models, were ultimately included in this review.

In the screening stage, the inter-rater agreement was calculated, and the *κ* value was 0.85 (95%CI: 0.78, 0.90), indicating a high degree of inter-rater agreement.

### 3.2 Study characteristics

Table 1 summarizes the design and participant details of the 14 reviewed studies. The studies, all conducted in China with 10 published in Chinese, span the years from 2017 to 2023. Among the included studies, 6 were prospective, 7 were retrospective, and 1 was a mixed retrospective and prospective study. There were 11 single-center studies and 3 multi-center studies. Regarding the study populations, 3 studies investigated severely ill patients without specifying a particular disease, 5 studies concentrated on patients with neurological disorders (including 3 studies on stroke patients), 2 studies targeted patients with severe acute pancreatitis, 3 studies focused on patients with severe sepsis, and 1 study examined patients with gastric cancer undergoing gastrectomy. The number of participants in each study varied between 118 and 628. Enteral nutrition (EN) was monitored for a duration ranging from a minimum of 5 days to a maximum of 2 weeks, with most studies focusing on 7 days. The incidence of FI in enteral nutrition was 32.4–63.1% in 14 studies.

**Table 1:** Overview of basic data of the included studies.

**Author (year)**	**Country**	**Study design**	**Participants**	**Follow-up time**	**Data source**	**Outcome index**	**FI cases/sample size (%)**
Wang et al. (26)	China	Mixed retrospective and prospective cohort study	ICU patient	Continuous assessment was performed for 7 days from the start of EN	Comprehensive ICU	Intolerance to enteral feeding for any clinical reason, such as vomiting, high gastric residual volume, diarrhea, gastrointestinal bleeding, enterocutaneous fistula, etc. The presence of FI should be considered if it is not possible to reach at least 20 kcal/kg/day via the enteral route within 72 h of attempted feeding or if EN must be discontinued for any clinical reason	308/628 (49.0%)
Yibo et al. (33)^a^	China	Prospective cohort study	Critically ill neurosurgical patient	Continuous assessment was performed for 5 days from the start of EN	Neurosurgery ICU	EN interrupts or pauses due to gastrointestinal symptoms such as diarrhea, abdominal distension, vomiting, reflux, and high gastric residual volume during EN, resulting in insufficient energy intake of 83.72 kJ/(kg·d) during the first 72 h of EN.	68/144 (47.2%)
Jinfeng (24)^a^	China	Retrospective cohort study	Patient with severe acute pancreatitis	—	Comprehensive ICU	(1) Termination or suspension of EN due to discomfort such as vomiting or reflux, abdominal distension, diarrhea, gastrointestinal bleeding, weakened or disappeared bowel sounds, constipation, and gastric residual volume ≥ 500 mL/24 h; (2) The patient did not reach the caloric target of 20 kJ/(kg·d) after 72 h of EN; FI can be diagnosed if any one of the above two factors is met	103/246 (41.9%)
Lihong et al. (25)^a^	China	Retrospective cohort study	Patients with sepsis in the ICU	Continuous assessment was performed for 7 days from the start of EN	Comprehensive ICU	(1) Gastric retention: ≥200 mL/24 h; (2) Vomiting: vomiting from the oropharynx or nasopharynx one or more times; (3) Diarrhea: watery stool ≥3 times within 24 h, each stool volume > 200 g; (4) Abdominal distension: intra-abdominal pressure ≥ 12 mmHg; (5) aspiration: oropharyngeal food, secretions or esophageal reflux into the subglottic airway; (6) Reflux: gastric contents reflux to the oropharynx, without nausea, retching and other symptoms. When multiple clinical manifestations and signs of intolerance occurred at the same time, the most predominant symptom was used as the outcome	69/140 (49.3%)
Lu et al. (31)	China	Prospective cohort study	ICU patient	From the start of EN to the diagnosis of FI; EN for more than 2 weeks; Transfer out of ICU; Gastric tube withdrawal or death	Comprehensive ICU, emergency ICU and neurosurgery ICU	Patients with one or more gastrointestinal symptoms that result in a reduction or suspension of EN within the first 2 weeks are diagnosed with FI	77/203 (37.93%)
Jiaxin et al. (27)^a^	China	Prospective case–control study	Patients with stroke	From the start of EN, the patients were continuously observed for 5 days for gastrointestinal symptoms, and the presence of one or more of the above symptoms within 24 h was defined as FI	Neurosurgery ICU	FI was determined by the working group of the European Society of Critical Care Medicine (ESICM)	51/118 (43.2%)
Wei (30)^a^	China	Prospective cohort study	Neurosurgical patient	—	Neurosurgery ICU	FI caused by various causes such as vomiting, gastric retention, diarrhea, gastrointestinal bleeding, enterocutaneous fistula, etc., can be diagnosed by meeting any of these factors	46/127 (36.2%)
Xiaoping (32)^a^	China	Prospective cohort study	ICU patient	Continuous assessment was performed for 7 days from the start of EN	Comprehensive ICU, emergency ICU and neurosurgery ICU	Patients had diarrhea, excess gastric residual, abdominal distension, constipation, vomiting and/or regurgitation, weakened or disappeared bowel sounds, and gastrointestinal bleeding	68/210 (32.4%)
Xiaolan et al. (22)^a^	China	Retrospective cohort study	Patient with Severe Stroke	Continuous assessment was performed for 7 days from the start of EN	Department of Geriatrics	Poor tolerance was defined as the patient who could continue EN after adjusting the speed and total amount of EN despite having one of the four symptoms, such as vomiting, diarrhea, gastric retention or aspiration. If any of the above symptoms could not be relieved after active treatment, the patients were considered as completely intolerant	107/282 (37.9%)
Guiying et al. (23)^a^	China	Retrospective cohort study	Patient with stroke	Within 7 days of initiation of EN	Department of Brain Disease	(1) GI symptoms and signs, including vomiting, bloating, diarrhea, constipation, and diminished or absent bowel sounds; (2) EN interruption due to various reasons; (3) high gastric residue (gastric residue ≥500 mL for 24 h). The occurrence of FI was defined as the occurrence of any of the above	76/206 (63.1%)
Ting (29)^a^	China	Prospective observational study	Adult patient with sepsis	The maximum number of days observed was specified to be 7 days from the initiation of EN	Comprehensive ICU	Patients with EN in the process of vomiting/reverse flow, abdominal distention, diarrhea, gastric residual volume 250 mL/6 or higher h, constipation, gastrointestinal bleeding and gastrointestinal adverse reactions, including aspiration of one or a variety of symptoms that is defined as FI	140/271 (51.7%)
Hu (28)	China	Retrospective case–control study	Adult patient with sepsis	—	Double center ICU	Patients presented with vomiting, abdominal distension, high gastric residual (gastric residual ≥500 mL/24 h), diarrhea, and high intra-abdominal pressure (intra-abdominal pressure > 12 mmHg) are diagnosed with FI	86/195 (44.1%)
Fuyan et al. (21)^a^	China	Retrospective cohort study	Patient with severe acute pancreatitis	—	Gastroenterology department	EN patients appeared in the process of gastrointestinal adverse reactions such as abdominal distention, diarrhea, constipation, EN to suspend or terminate, in patients within 72 h cannot achieve 83.68 kJ/(kg · d) target of heat	49/118 (41.9%)
Xiaoyong et al. (20)	China	Retrospective cohort study	Gastric cancer gastrectomy patient	EN is maintained for at least 5 days	Gastroenterology department	Diarrhea (>4 watery stool or stool volume ≥ 200 mL/24 h); Vomiting; Abdominal bloating; High stomach residue (stomach residue ≥473 mL/24 h)	111/225 (49.30%)

a Study was published in Chinese.

ICU, Intensive Care Unit; EN, Enteral Nutrition; FI, Feeding Intolerance; GI, Gastrointestinal.

In the data extraction stage, the results of inter-rater agreement were calculated as follows: for binary or nominal variables, the unweighted Cohen’s kappa (*κ*) value was 0.82 (95% CI: 0.75, 0.91), showing good inter-rater agreement; for continuous variables, two-way intraclass correlation coefficients (ICCs) were calculated, and the results showed that the ICCs value was 0.90 (95% CI: 0.85, 0.94), which also indicated a high degree of inter-rater agreement.

### 3.3 Results of quality assessment

**Figure 2:**
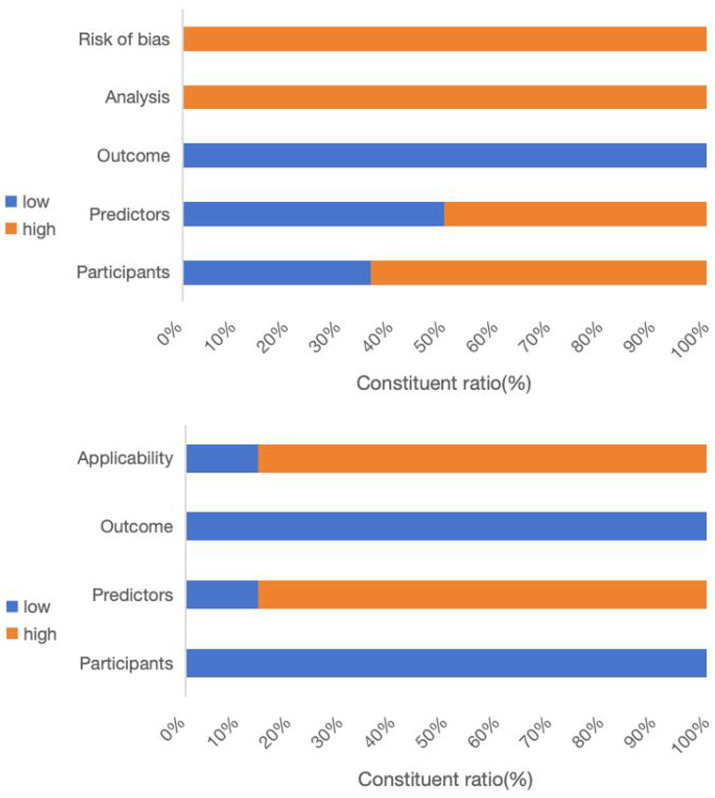
Bar chart of PROBAST bias risk and suitability evaluation results for included studies.

All studies incorporated in this systematic review were evaluated using the GRADE approach to ensure high certainty in their conclusions. Table 2 and Figure 2 outline the risks of bias and applicability in the included studies.

**Table 2:** PROBAST results of the included studies.

Author(year)	ROB				Applicability			Overall	
	Participants	Predictors	Outcome	Analysis	Participants	Predictors	Outcome	ROB	Applicability
Wang et al. (26)	−	+	+	−	+	+	+	−	+
Yibo et al. (33)^a^	+	+	+	−	+	+	+	−	+
Jinfeng (24)^a^	−	−	+	−	+	+	+	−	+
Lihong et al. (25)^a^	−	−	+	−	+	+	+	−	+
Lu et al. (31)	+	+	+	−	+	+	+	−	+
Jiaxin et al. (27)^a^	−	+	+	−	+	+	+	−	+
Wei (30)^a^	+	+	+	−	+	+	+	−	+
Xiaoping (32)^a^	+	+	+	−	+	+	+	−	+
Xiaolan et al. (22)^a^	−	−	+	−	+	+	+	−	+
Guiying et al. (23)^a^	−	−	+	−	+	+	+	−	+
Ting (29)^a^	+	+	+	−	+	+	+	−	+
Hu (28)	−	−	+	−	+	+	+	−	+
Fuyan et al. (21)^a^	−	−	+	−	+	−	+	−	−
Xiaoyong et al. (20)	−	−	+	−	+	−	+	−	−

PROBAST, Prediction model risk of bias assessment tool; ROB, risk of bias. + indicates low ROB/low concern regarding applicability; − indicates high ROB/high concern regarding application^a^Study was published in Chinese.

Upon conducting a model bias risk assessment, it was determined that all studies exhibited a significant level of bias, suggesting the presence of methodological flaws in their development or validation stages. About the participant domain, the risk prediction model for feeding intolerance in patients with enteral nutrition is a prognostic prediction model study. In such cases, a prospective cohort study is generally recommended. Of the studies analyzed, 9 were identified as having a high risk of bias. These included 6 retrospective cohort studies (20–25), 1 prospective and retrospective cohort study (26), 1 prospective case–control study (27), and 1 retrospective case–control study (28). Within the predictor domain, 7 studies were identified to have a significant risk of bias (20–25, 28). These 7 studies were retrospective, and none of them addressed the matter of blinding. Consequently, the measurement process was susceptible to being influenced by pre-existing outcomes, leading to an elevated risk of bias. In terms of the outcome domain, all the studies incorporated were deemed to possess a low risk of bias. In the analysis field, the sample size of prediction model studies was more concerned with the number of subjects with predicted outcomes. Among them, 6 studies exhibited a substantial risk of bias when the ratio of participants to potential predictors fell below 10 (21, 25, 27, 29–31). The continuous variables of 7 studies were not processed (20, 26, 28–32), and the continuous variables of data in 1 study were temporarily converted to binary variables for prediction in the analysis stage (27), which were all considered as high risk of bias. Twelve studies failed to address the management of missing data, thereby posing a significant risk of bias (20–25, 28–33). A single study utilized a machine learning algorithm to reduce the bias stemming from univariate analysis, whereas the other studies were assessed to pose a significant risk of bias (28). All studies did not mention the treatment of complex issues in the data and did not consider or mention the overfitting or underfitting of the relevant models, and were therefore considered to have a high risk of bias (20–33).

Regarding applicability evaluation, just 2 studies were assessed as having limited applicability, whereas the majority showed good applicability. In the area of participants and outcomes, all studies demonstrated a low risk of bias. 2 studies within the predictor domain were identified as having a high risk of applicability concerns, primarily due to issues pertaining to the timing of the predictor assessments (20, 21).

### 3.4 Construction/validation of included models

Table 3 offers a summary of the study-included model details. The 14 included studies reported 18 FI prediction models for enteral nutrition. In terms of data preprocessing, two studies stated that missing data were eliminated directly, but the number of missing values was not reported (26, 27). None of the other 12 studies mentioned the preprocessing methods of the data. In terms of model construction, only one study used multiple machine learning methods to build a model and then selected the best model according to the model evaluation index (28). The remaining studies only used a single method for modeling, and the modeling method was univariate and multivariate Logistic regression. In terms of model validation, 8 studies only conducted internal validation (20, 21, 23, 25, 27, 29–31), and the remaining 6 studies used a combination of internal and external validation to evaluate the model (22, 24, 26, 28, 32, 33).

**Table 3:** Overview of the information of the included prediction models.

Author (year)	Missing data handling	Continuous variable processing method	Variable selection	Sample size		Model development method	Calibration method	Validation method	Final predictors	Model performance		Model presentation
				Modeling group	Verification group					Modeling group	Verification group	
Wang et al. (26)	The missing key indicators were eliminated	Continuous variable	Stepwise regression analysis (backward LR method)	628	143	LR	Hosmer-Lemeshow goodness of fit test, Calibration curve	Internal verification External validation	The main diagnoses were circulatory disease, APACHE II score, and AGI grade	A: 0.850 (0.821–0.879)	B: 0.879 (0.823–0.936)	Nomogram model
Yibo et al. (33)^a^	—	Categorical variables	—	144	61	Lasso regression analysis	Hosmer-Lemeshow goodness of fit test, Calibration curve	Internal verification External validation	Mean arterial pressure, combined use of more than 2 antibiotics, GCS score, intake and output volume	A:0.869 (0.810–0.928)	B:0.816 (0.711–0.920)	Nomogram model
Jinfeng (24)^a^	—	Categorical variables	—	246	105	LR	Hosmer-Lemeshow goodness of fit test, Calibration curve	Internal verification External validation	Hypertriglyceridemia, hypoproteinemia, intra-abdominal pressure ≥ 12 mmHg, APACHEII score ≥ 20, time to start enteral nutrition ≥72 h, addition of microecological agents	A:0.793 (0.735–0.851)	B:0.888 (0.822–0.954)	Nomogram model
Lihong et al. (25)^a^	—	Categorical variables	—	140	—	LR	Calibration curve	Internal verification: bootstrap resampling method	APACHE II score, mNutric score, CRRT, intra-abdominal pressure ≥ 12 mmHg, low calorie energy	A: 0.906 (0.783–1.000)	—	Nomogram model
Lu et al. (31)	—	Continuous variable	Stepwise regression analysis	203	—	LR	Calibration curve	Internal verification: bootstrap resampling method	Age, Gastrointestinal disease, Early feeding, Mechanical ventilation before enteral nutrition, Abnormal serum sodium	A: 0.700	—	Nomogram model
Jiaxin et al. (27)^a^	The missing key indicators were eliminated	Continuous variable	Stepwise regression analysis	118	—	LR	Calibration curve	Internal verification: bootstrap resampling method	Age, APACHE II score, bed time, albumin, vasoactive drugs, bedside Angle ≥30°	C-index = 0.879	—	Nomogram model
Wei (30)^a^	—	Continuous variable	—	127	—	LR	Calibration curve	Internal verification	Hypertension, mechanical ventilation, sedative and analgesic drugs, hyperkalemia, hyperglycemia, length of ICU stay, low Glasgow coma index score	A: 0.889 (0.821–0.938)	—	Nomogram model
Xiaoping (32)^a^	—	Continuous variable	Stepwise regression analysis	210	105	LR	Hosmer-Lemeshow goodness of fit test, Calibration curve	Internal verification: bootstrap resampling method External validation	APACHEII score, NRS2002 score, intra-abdominal pressure, albumin and fasting blood glucose	A: 0.921 (0.885–0.958)	B: 0.972 (0.943–0.996)	Prediction probability equation
Xiaolan et al. (22)^a^	—	Categorical variables	Stepwise regression analysis	282	—	LR	Hosmer-Lemeshow goodness of fit test, Calibration curve	Internal verification: bootstrap resampling method External validation	Age ≥ 60 years old, use of more than 2 kinds of antibacterial drugs, use of probiotics, and mechanical ventilation	A: 0.794 (0.741–0.847)	B: 0.764 (0.690–0.839)	An online visualized dynamic nomogram was generated based on the DynNom program
Guiying et al. (23)^a^	—	Categorical variables	Stepwise regression analysis	206	—	LR	Calibration curve	Internal verification	Antacids, mechanical ventilation, age, and NIHSS score	A: 0.773 (0.679–0.851)	—	Prediction probability equation
Ting (29)^a^	—	Continuous variable	Stepwise regression analysis	271	—	LR	Hosmer-Lemeshow goodness of fit test, Calibration curve	Internal verification: bootstrap resampling method	AGI grading, start enteral nutrition, enteral nutrition, the average time infusion speed, C - reactive protein, albumin, early enema, adding glutamine	A: 0.885 (0.845–0.921)	—	Nomogram model
Hu (28)	—	Continuous variable	—	124	71	LR, NB, RF, GBT, DL	Calibration curve	Internal verification: five-fold cross-validation External validation	Twenty-seven, the top five items of relative importance were pulmonary infection, nutritional type, shock, skin infection, and continuous feeding	LR A: 0.73 (0.64–0.82) NB A: 0.70 (0.60–0.80) RF A: 0.92 (0.87–0.97) GBT A: 0.94 (0.90–0.99) DL A: 0.82 (0.74–0.90)	LR B: 0.69 (0.56–0.81) NB B: 0.73 (0.60–0.85) RF B: 0.63 (0.50–0.76) GBT B: 0.60 (0.47–0.74) DL B: 0.79 (0.68–0.89)	web online prediction tool
Fuyan et al. (21)^a^	—	Categorical variables	—	118	—	LR	Calibration curve	Internal verification: bootstrap resampling method	Age ≥ 70 years old, Fasting blood glucose ≥11.mmol/L, The starting time of the enteral nutrition ≥72 h, Not adding dietary fiber, Intra-abdominal pressure ≥ 15 mmHg	C-index = 0.869 A: 0.857 (0.779–0.931)	—	Nomogram model
Xiaoyong et al. (20)	—	Continuous variable	Backward likelihood ratio method	225	—	LR	Hosmer-Lemeshow goodness of fit test, Calibration curve	Internal verification	Secret history of stool, Preoperative ASA score was level III, The pain score at 6 h after surgery was high, The white blood cell count was high on the first day after surgery	A: 0.756	—	Nomogram model

“—,” not reported; A, development cohort; B, validation cohort; LR, Logistic Regression; NB, Naive Bayesian; RF, Random Forest; GBT, Gradient Boosting Tree; DL, Deep Learning, NIHSS: National Institutes of Health Stroke Scale.^a^Study was published in Chinese.

### 3.5 Performance and predictors of the included models

The discrimination of 14 studies was reported using the area under the ROC curve, with values spanning from 0.70 to 0.921. Regarding the calibration methods employed for the model, seven studies exclusively utilized the calibration curve (21, 23, 25, 27, 28, 30, 31), and the other studies used the calibration curve with Hosmer-Lemeshow goodness of fit to evaluate the calibration degree (20, 22, 24, 26, 29, 32, 33). The models were mainly presented as prediction probability equations and nomograms. Two studies presented the results in the form of prediction probability equations (23, 32), 10 studies presented the results in the form of static nomograms (20, 21, 24–27, 29–31, 33), and 2 studies presented the results in the form of online dynamic nomograms (22, 28).

**Figure 3:**
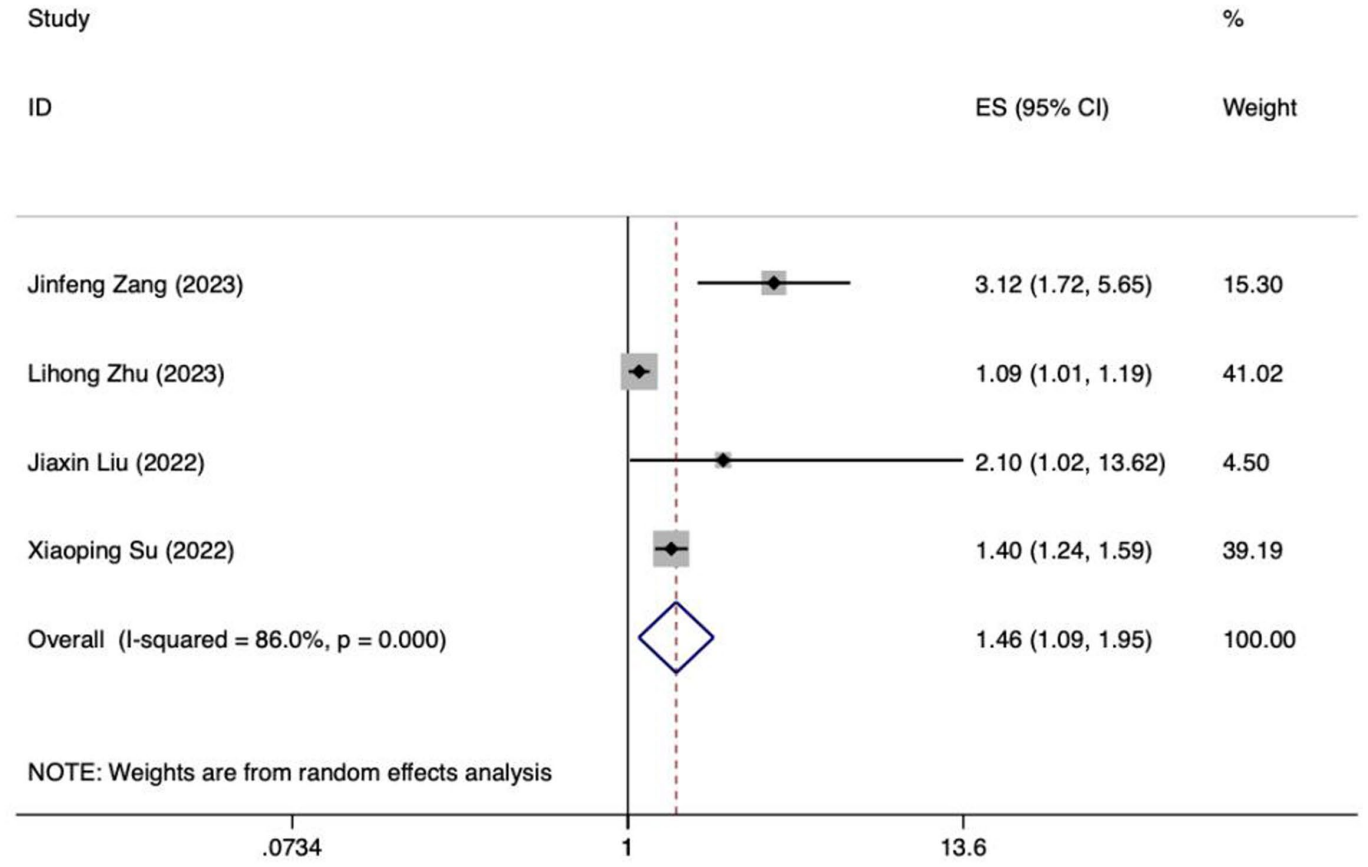
Forest plot of meta-analysis of APACHE II score in predicting FI in patients with enteral nutrition.

**Figure 4:**
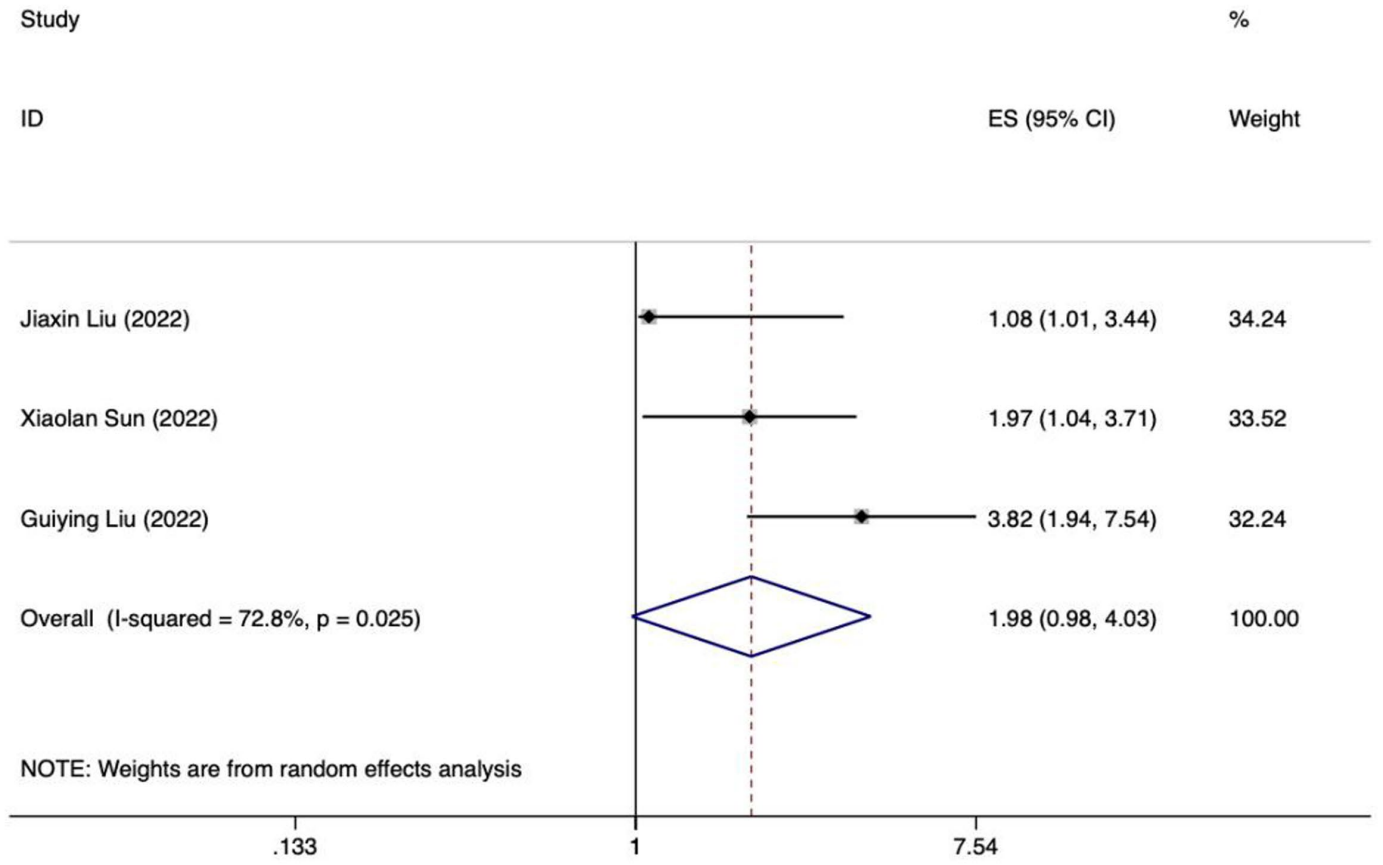
Forest plot of meta-analysis of age in predicting FI in patients with enteral nutrition.

**Figure 5:**
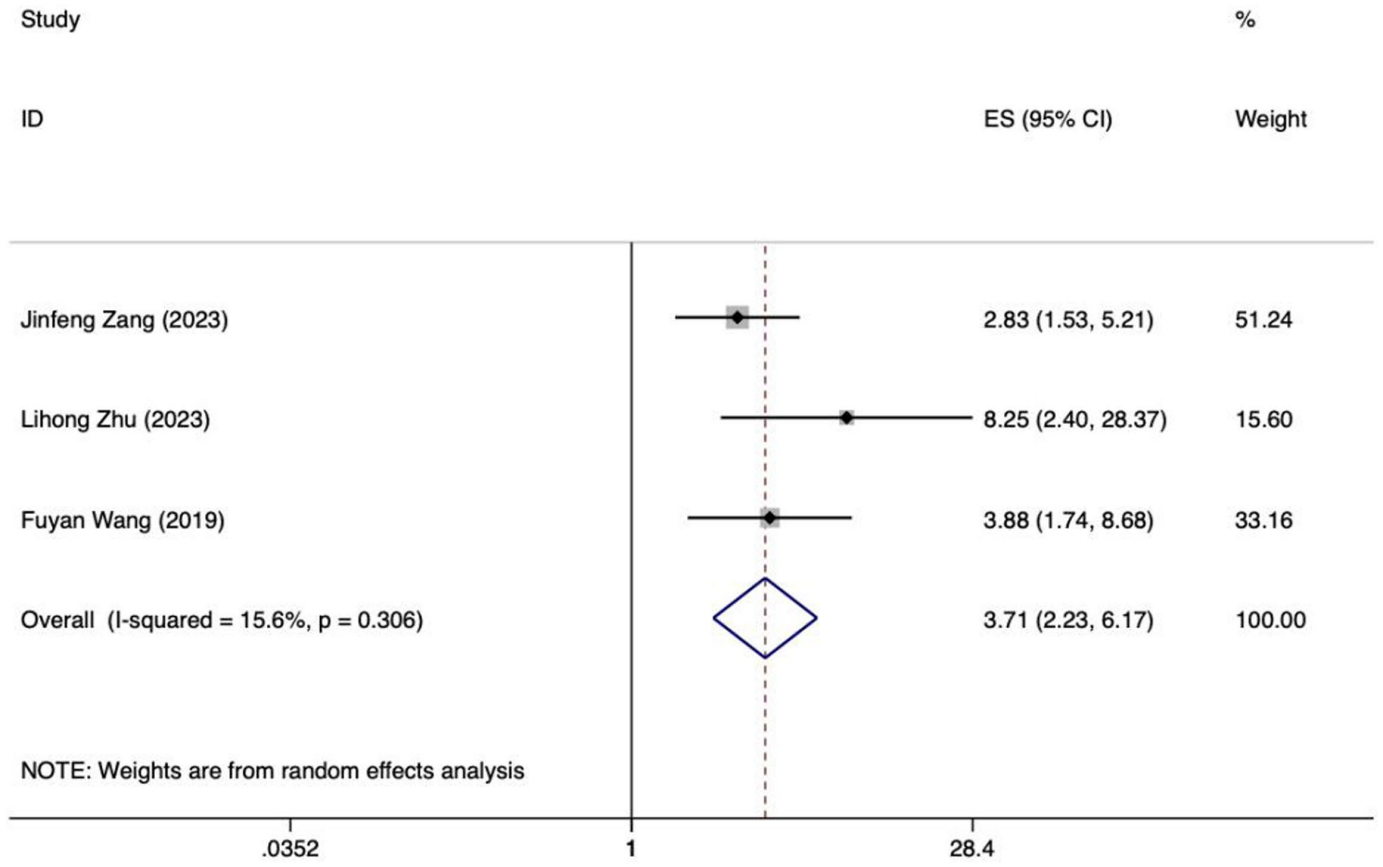
Forest plot of meta-analysis of albumin levels in predicting FI in patients with enteral nutrition.

**Figure 6:**
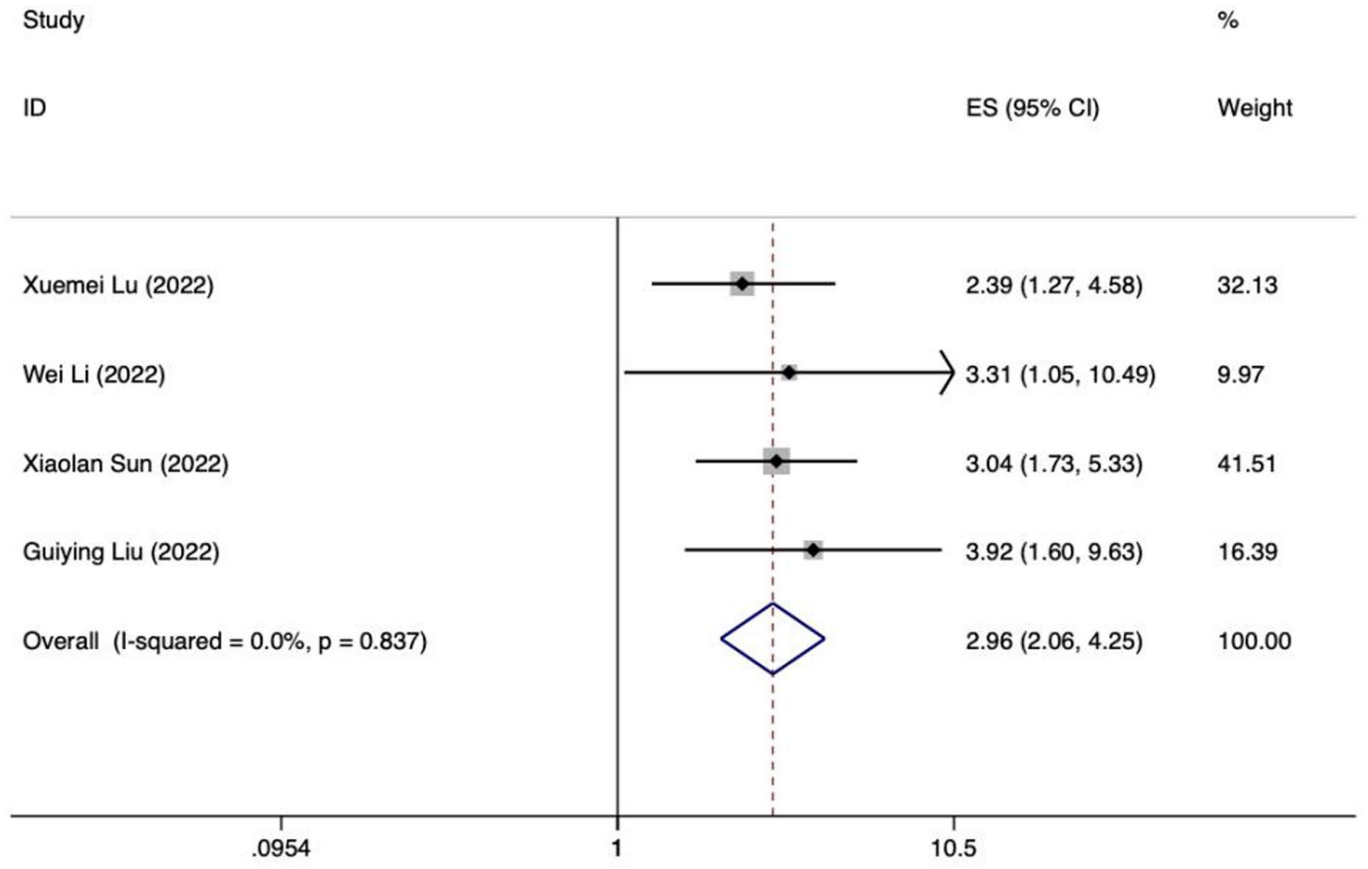
Forest plot of meta-analysis of intra-abdominal pressure predicting FI in patients with enteral nutrition.

**Figure 7:**
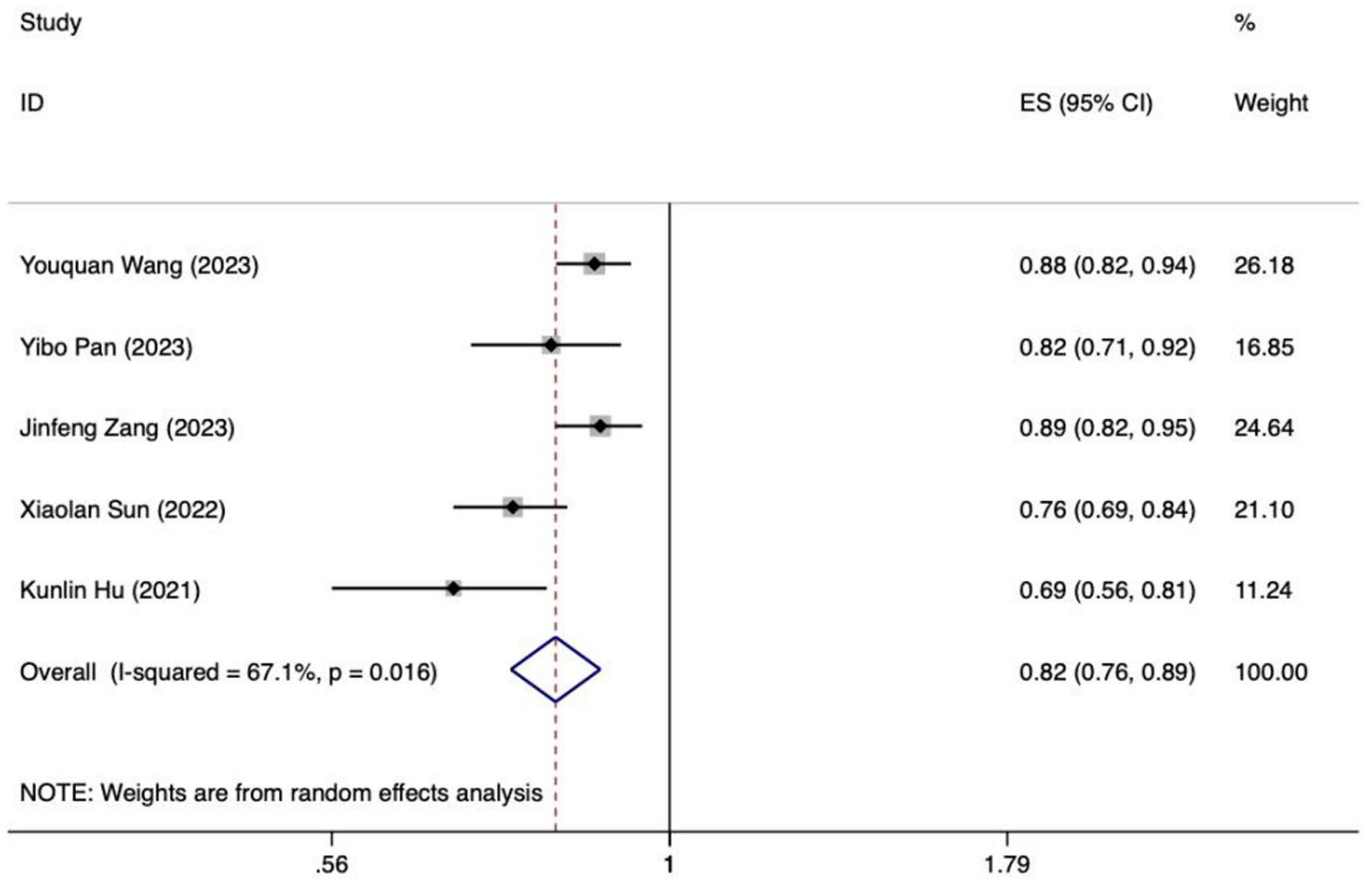
Forest plot of meta-analysis of mechanical ventilation for predicting FI in patients with enteral nutrition.

The present study encompasses a total of 14 articles and 18 models. Hu and their team created five different models, employing Logistic regression, the Naive Bayes method, the Random Forest algorithm, the Gradient Boosting Tree technique, and a Deep Learning strategy, respectively (28). The maximum number of predictors included in each model was 27, and the minimum was 3. The most frequently employed predictors in each model are as follows: age and APACHE II score, which appeared in 5 models, respectively. Other commonly used predictors included albumin, intra-abdominal pressure and mechanical ventilation, each for the 4 models. Hyperglycemic, addition of microecological agents, time to start enteral nutrition and nutritional scores were used in each of the 3 included models. In addition, acute GI injury grading, GCS score, and combined use of antibiotics were used in the two models included. The predictive effect of the top five predictors on FI in patients with enteral nutrition was analyzed, and the results are shown in Table 4, the forest plots were shown in Figures 3–7.

**Table 4:** Meta-analysis results of the top 5 predictors.

Predictor	Model of effect	Pooled effect size				Test of heterogeneity	
		*OR*	*95%CI*	Value of *Z*	Value of *P*	*I^2^*(%)	Value of *P*
APACHE II	Random	1.46	1.09–1.95	2.53	0.011	86.0	<0.001
Age	Random	1.98	0.98–4.03	1.89	0.058	72.8	0.025
Albumin levels	Random	0.97	0.84–1.13	0.35	0.724	73.3	0.010
Intra-abdominal pressure	Fixed	3.71	2.23–6.17	5.06	<0.001	15.6	0.306
Mechanical ventilation	Fixed	2.96	2.06–4.25	5.85	<0.001	0	0.837

Meta analysis of APACHE II score for predicting enteral nutrition FI. The sensitivity analysis was conducted using the stepwise literature elimination method. The results demonstrated a notable discrepancy in the combined effect size observed in the study conducted by Wang et al., which may be a contributing factor to the observed heterogeneity. As a result, this study was omitted from the analysis process (26). A meta-analysis was performed on the 4 remaining articles (24, 25, 27, 32), and the heterogeneity among the studies was high (*I^2^* = 86.0%, *p* < 0.001). A random-effects model was utilized for the purpose of combining the effect sizes. The results demonstrated a statistically significant discrepancy (*OR* = 1.46, 95%*CI*: 1.09–1.95, *p* = 0.011). The Egger test yielded a t-value of 1.60 and a *p*-value of 0.250, indicating the absence of significant publication bias in the results. Consequently, the included studies can be considered relatively reliable and less affected by publication bias.

Meta analysis of age for predicting enteral nutrition FI. The sensitivity analysis was conducted using the stepwise literature elimination method. The results showed significant differences in the combined effect size between Lu et al. and Wang et al., which may be a factor leading to heterogeneity, and were excluded (21, 31). Meta analysis was conducted on the remaining 3 articles (22, 23, 27), and the heterogeneity between the studies was high (*I^2^* = 72.8%, *p* = 0.025). A random-effects approach was used to pool the effect sizes together. The findings indicated no statistically notable difference (*OR* = 1.98, 95%*CI*: 0.98–4.03, *p* = 0.058).

Meta analysis of albumin levels for predicting enteral nutrition FI. The sensitivity analysis, performed through the sequential exclusion of literature, revealed no substantial variation in effect size. This underscores the stability and reliability of the meta-analysis findings. Meta analysis was conducted on the 4 included literatures (24, 27, 29, 32). The heterogeneity between the studies was high (*I^2^* = 73.3%, *p* = 0.010). For the purpose of combining the effect sizes, a random-effects model was utilized. The results demonstrated no statistically significant discrepancy (*OR* = 0.97, 95%*CI*: 0.84–1.13, *p* = 0.724).

Meta analysis of intra-abdominal pressure for predicting enteral nutrition FI. The sensitivity analysis was conducted using the stepwise literature elimination method. The results showed that Su et al.’s study had significant differences in the amount of combined effects, which may be a factor leading to heterogeneity, and the study was excluded (32). Meta analysis was conducted on the remaining 3 articles (21, 24, 25), and the heterogeneity between the studies was low (*I^2^* = 15.6%, *p* = 0.306). A fixed-effects model was utilized for the purpose of combining the effect sizes. The results demonstrated a statistically significant discrepancy (*OR* = 3.71, 95%*CI*: 2.23–6.17, *p* < 0.001). The results of the Egger test, with a t-value of 48.21 and a *p*-value of 0.013, suggested the absence of notable publication bias. This suggests that the included studies were relatively reliable and less affected by publication bias.

Meta analysis of mechanical ventilation for predicting enteral nutrition FI. The sensitivity analysis, which involved progressively excluding literature, showed no notable difference in effect size. This confirms the robustness and reliability of the meta-analysis findings. The 4 selected studies underwent meta-analysis, revealing low heterogeneity among them (*I^2^* = 0%, *p* = 0.837) (22, 23, 30, 31). The fixed-effects model was used to aggregate the effect sizes, producing statistically meaningful outcomes (*OR* = 2.96, 95%*CI*: 2.06–4.25, *p* < 0.001). The Egger test indicated no significant publication bias, with a t-value of 0.87 and a *p*-value of 0.477. Therefore, the included studies can be deemed relatively trustworthy and less influenced by publication bias.

### 3.6 Meta-analysis of validation models included in the review

**Figure 8:**
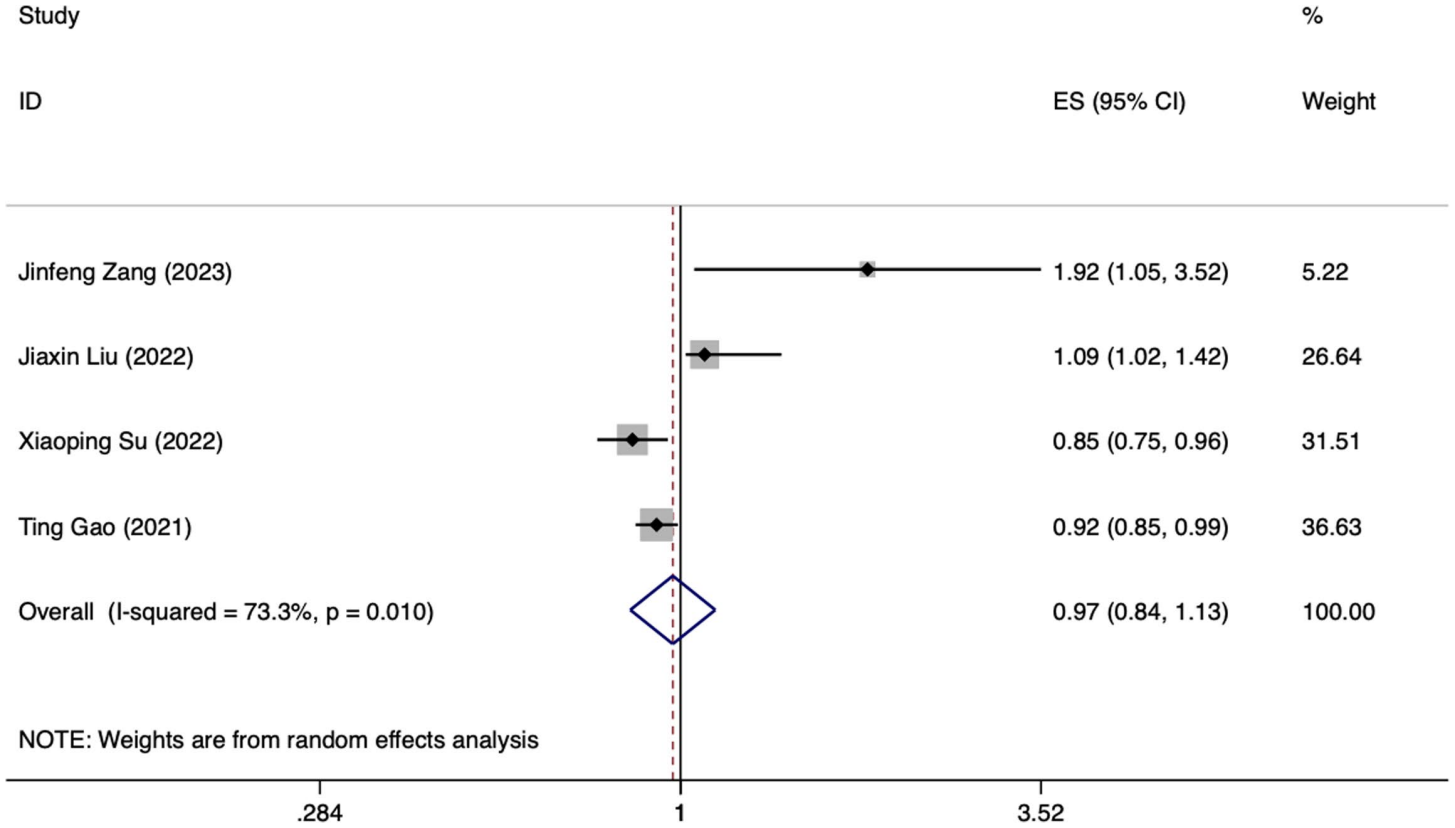
Forest plot of the random effects meta-analysis of pooled AUC estimates for 5 validation models.

Because of inadequate disclosure of model development specifics in the studies considered, only six studies met the criteria for synthesis. Included among these was the research by Hu et al., which utilized various methods and constructed a model employing logistic regression (28). Based on the sensitivity analysis results, if there was a significant decrease in heterogeneity or a notable change in the pooled effect size after excluding the study by Xiaoping Su et al., it would indicate that this study could be a major contributor to the heterogeneity (32). After discussion, it was decided to exclude certain literature, resulting in the inclusion of ultimately 5 studies in the meta-analysis (22, 24, 26, 28, 33). The *I^2^* value was 67.1%, indicating a significant degree of heterogeneity. A random-effects model was employed to compute the combined AUC, yielding a result of 0.82 (95%*CI*: 0.76–0.89, *p* < 0.001) (Figure 8). The AUC values ranged between 0.8 and 0.9, indicating a high level of predictive ability and stability for the overall model. Additionally, the Egger test produced a t-value of −2.73 and a *p*-value of 0.072, suggesting the absence of significant publication bias.

## 4 Discussion

### 4.1 Clinical implications of FI prediction models

The clinical implications of the prediction models discussed in this review are specific and significant. Despite conducting sensitivity analyses and excluding studies with high heterogeneity (e.g., APACHE II score, age, albumin levels, validation models), significant heterogeneity remained in our Meta-analysis results. This could be attributed to various factors, including differences in sample characteristics, research methodologies, and study quality among the remaining studies. The predictors highlighted in these models can also serve as focal points for future research. By investigating these factors in greater depth, researchers can gain a more comprehensive understanding of the underlying mechanisms that drive the development of various conditions, which can ultimately lead to the discovery of new and innovative treatments and therapies.

#### 4.1.1 APACHE II score

The study revealed that a high APACHE II score during enteral nutrition represented a significant risk factor for the development of FI. The APACHE II scoring system encompasses a variety of physiological indicators, such as body temperature, heart rate, blood pressure levels, and respiratory frequency. The abnormality of these parameters may directly reflect the gastrointestinal function status of patients, and then affect the tolerance of enteral nutrition (34). For example, hypotension may lead to hypoperfusion of the intestine, affecting the absorption and utilization of nutrients. Patients with high APACHE II score are often more serious, and their gastrointestinal function may be greatly affected, resulting in reduced tolerance to enteral nutrition (35). Severe trauma, sepsis and other patients may be accompanied by gastrointestinal dysfunction, which makes it difficult to effectively absorb and utilize enteral nutrition. Research has shown that ICU patients on mechanical ventilation who undergo enteral nutrition and have an APACHE II score of 20 or above are at increased risk of developing feeding intolerance (36). This implies that as the score rises, the likelihood of patients experiencing intolerance to enteral nutrition also increases.

#### 4.1.2 Intra-abdominal pressure

Various factors can contribute to elevated intra-abdominal pressure, including trauma, abdominal surgical procedures, inflammation, and extensive fluid resuscitation. In critically ill patients, intra-abdominal pressure is often elevated due to increased vascular permeability, inflammatory transmitter release, and fluid resuscitation (37). Increased intra-abdominal pressure will directly compress the intestine, inhibit gastrointestinal emptying and peristalsis function, and lead to decreased intestinal absorption capacity. This can affect the digestion and absorption of enteral nutrients, thereby increasing the risk of feeding intolerance of enteral nutrition (38). A number of studies have demonstrated that elevated intra-abdominal pressure is a significant risk factor for feeding intolerance associated with enteral nutrition (39–41). When the intra-abdominal pressure rises to a certain degree (such as ≥15 mmHg), the feeding intolerance will increase significantly.

#### 4.1.3 Mechanical ventilation

The findings indicated that mechanical ventilation during enteral nutrition constituted a significant risk factor for FI. This correlation may stem from the physiological impact of the positive end-expiratory pressure (PEEP) administered by the ventilator. Specifically, PEEP is known to elevate intrathoracic pressure, which in turn can impede venous return to the heart, thereby reducing cardiac output. Consequently, this reduction in blood flow may lead to insufficient perfusion of the mesenteric artery, ultimately impacting intestinal function and exacerbating the risk of FI (42). Qin Ming et al. also showed that positive end-expiratory pressure was positively correlated with the occurrence of FI in mechanically ventilated patients (43). Their findings reinforce the notion that careful monitoring and management of mechanical ventilation parameters, particularly PEEP, are crucial in mitigating the risk of FI among critically ill patients receiving enteral nutrition.

It is recommended that medical staff pay close attention to people at high risk of FI who are high APACHE II score, with high intra-abdominal pressure or undergoing mechanical ventilation, and regularly assess their risk of FI. For patients with high APACHE II score, medical staff should pay more attention to their gastrointestinal function and regularly assess enteral nutrition tolerance, so as to detect and deal with feeding intolerance in time (44). For patients receiving enteral nutrition support, the level of IAP should be monitored regularly, so that the situation of elevated IAP can be detected and treated in time. For patients with elevated IAP, medical staff should adjust the infusion plan of enteral nutrition individually according to the specific condition of the patient and the level of IAP (45). For example, in patients with IAP >15 mmHg, enteral nutrition support may be provided through a nasojejunal tube, and the rate of infusion may be gradually increased according to tolerance (46). The risk factors that can be intervened should be controlled, such as assessing the necessity of mechanical ventilation daily and assisting patients to be weaned from mechanical ventilation as soon as possible under the premise of ensuring treatment. Research has shown that abdominal massage can effectively alleviate gastric retention, abdominal bloating, aspiration risks, gastric residual volume, and reduce abdominal circumference in patients undergoing mechanical ventilation (47). For patients with non-interventionable risk factors, it is necessary to prospectively add parenteral nutrition and control the speed and amount of enteral nutrition intake to reduce the occurrence of FI and the incidence of malnutrition.

### 4.2 Evaluation and bias of FI prediction models

The development process of the included models offers valuable insights. The ROC curve areas for the 14 studies incorporated in this research varied between 0.70 and 0.921, which implies that the models involved demonstrated satisfactory predictive accuracy and were capable of precisely identifying patients experiencing FI related to enteral nutrition. However, the findings of the bias risk assessment revealed a considerable potential for bias in all studies. The underlying causes were identified as follows: In terms of study populations, some research data originated from retrospective cohort studies or classical case–control studies, potentially leading to bias. Within the context of research predictors, the heightened risk of bias arises from the considerable likelihood of bias in collecting predictors, chiefly because blinding is often absent in retrospective studies. Some predictors used in the included studies (e.g., microecological agents, early feeding) lack adequate theoretical support or evidence from the literature. We have emphasized that while these predictors may have potential clinical relevance, their inclusion in prediction models should be approached with caution due to the lack of robust evidence. Within the scope of research analysis, all studies were evaluated as having a significant potential for bias, largely stemming from the pervasive problem of missing data or flawed analytical procedures. In summary, some studies rely on limited sample sizes and are based on single-center retrospective research, with a notable absence of external validation. On the one hand, there are regional limitations of single-center data, and its representativeness and popularization need to be investigated. Research data derived from existing data or retrospective data, and insufficient sample size for model construction will increase the risk of bias of the model to a certain extent. On the other hand, the importance of external validation, particularly in a multicenter context, cannot be overstated. Multicenter validation ensures that models are tested across a broader range of patient demographics, clinical practices, and institutional protocols. This approach enhances the robustness and reliability of the models, making them more applicable and trustworthy in various healthcare settings. Furthermore, among the 14 included studies, only one utilized machine learning methods for modeling, while the rest primarily relied on logistic regression. Both methods have their respective strengths and weaknesses. Logistic regression, as a classical statistical method, is widely used due to its strong interpretability, simplicity in calculation, and fast training speed. It is particularly suitable for handling binary classification problems and can directly provide probability outputs, which are easy to understand and interpret. However, logistic regression may struggle when fitting complex, multidimensional data, especially when the data exhibits nonlinear relationships, missing values, or multicollinearity, which can affect its predictive performance. In contrast, machine learning techniques, especially modern methods such as deep learning, demonstrate robust capabilities in handling large-scale, high-dimensional, and complex data. They can automatically learn the underlying patterns and regularities in the data without requiring manually specified functional forms. Therefore, machine learning models typically fit the true distribution of the data better and exhibit higher predictive accuracy on new data. Additionally, machine learning techniques can handle issues such as missing values and multicollinearity, further enhancing the robustness and generalization ability of the models (48). Hu et al. (28) utilized five distinct machine learning techniques: logistic regression, the naive Bayes classifier, the random forest algorithm, the gradient boosting tree method, and deep learning, to create FI risk prediction models tailored specifically for sepsis patients. The assessment of the models’ predictive accuracy involved computing the area enclosed by the receiver operating characteristic curve. In the validation dataset, the deep-learning model excelled with an AUC value of 0.79, whereas logistic regression lagged behind with an AUC of just 0.69, suggesting its inferior predictive capabilities relative to other machine-learning techniques. Nonetheless, there was a substantial amount of heterogeneity among the models, potentially resulting from variations in the demographic characteristics, choice of predictors, and the specific methodologies employed by each. However, it is worth noting that despite their superior predictive performance, machine learning models are often more complex and less interpretable. Additionally, the model construction and training process can be influenced by various factors, such as data preprocessing methods, feature selection strategies, and algorithm parameter settings. Therefore, when applying machine learning techniques, it is necessary to carefully select algorithms and parameters and conduct sufficient validation and tuning to ensure the reliability and stability of the models. Moreover, upon reviewing the models, it was discovered that several studies lacked adequate adherence to the guidelines outlined in the Transparent Reporting of a Multivariate Prediction Model for Individual Prognosis or Diagnosis (TRIPOD) statement. The resulting lack of transparency introduces ambiguity and heightens the risk of bias within the models. Therefore, it is recommended that future research focus on creating new models with expanded sample sizes, stringent research methodologies, multi-center external validations, and improved clarity in reporting to enhance transparency.

## 5 Limitations

It should be noted that this review is subject to a number of potential limitations. Firstly, it is worth noting that all studies considered were undertaken solely in mainland China, potentially limiting the broader applicability of the findings to Western populations. As a result, adjustments may be necessary when applying these models to various regions. Accordingly, the development of risk prediction models tailored to various populations is of significant importance for future research endeavors. Secondly, owing to variations in the predictors utilized by the included prediction models, our meta-analysis incorporated solely the top five predictors. Given the limited number of predictors encompassed in the studies, the statistical robustness of the meta-analysis results may be insufficient to substantiate the reliability of the drawn conclusions. In addition, statistical analysis methods such as heterogeneity test and sensitivity analysis in meta-analysis may also be biased due to differences between studies. Lastly, it should be noted that the present review encompasses only studies published in English and Chinese, which may introduce potential limitations in terms of language bias and the generalizability of its findings.

## 6 Conclusion

A total of 14 articles, encompassing 18 risk prediction models for feeding intolerance (FI) in patients receiving enteral nutrition, were incorporated into the review. The incidence of FI in patients with enteral nutrition was 32.4–63.1%. The top five predictors included in the model were APACHE II score, age, albumin level, intra-abdominal pressure and mechanical ventilation. According to the PROBAST framework, all studies incorporated in the analysis were identified as posing a notable risk of bias, and concerns regarding applicability were raised for two studies in particular. The existing prediction models for feeding intolerance (FI) in patients undergoing enteral nutrition do not fulfill the standards set by PROBAST. Hence, it is crucial for researchers to thoroughly familiarize themselves with the PROBAST checklist and adhere to the reporting guidelines stipulated in the TRIPOD statement, in order to enhance the quality of their future research endeavors.

In future research endeavors, it is imperative to adopt a more tailored approach in developing a representative and widely applicable FI risk prediction model. Specifically, this involves selecting appropriate machine learning algorithms, such as ensemble methods or deep learning frameworks, which have shown promise in handling complex and large datasets. Additionally, utilizing substantial sample sizes will be crucial to ensure the robustness and generalizability of the model. Moreover, designing multi-center studies with rigorous methodologies is essential. This can be achieved by collaborating with multiple institutions, establishing standardized protocols for data collection and analysis, and ensuring consistency in the definition and measurement of FI and its associated risk factors. To control bias, we should employ strategies such as randomization, blinding, and appropriate statistical adjustments. By taking these specific improvement measures, we can facilitate early identification of high-risk populations and streamline the implementation of targeted preventive interventions. Ultimately, this will help decrease the risk of FI and improve clinical outcomes for patients requiring enteral nutrition.

## Data Availability

The original contributions presented in the study are included in the article/Supplementary material, further inquiries can be directed to the corresponding author.
