# Supplementary material for: Risk prediction models for feeding intolerance in patients with enteral nutrition: a systematic review and meta-analysis
Source: Front Nutr. 2025 Jan 14;11:1522911. doi: 10.3389/fnut.2024.1522911 (PMC11772164; doi:10.3389/fnut.2024.1522911)
Supplement: Supplementary file 1 [file Supplementary_file_1.docx]

Supplementary Material

# Supplementary table

Table 1

Database and search Strategy.

| Database | Retrieval formula | Search results |
| --- | --- | --- |
| CNKI | FT=喂养不耐受 AND FT=预测模型 | 213 |
| Wanfang Data | 全部:(喂养不耐受 or 肠内营养) and 全部:(预测模型) | 77 |
| VIP | (M=肠内营养 OR 喂养不耐受) AND M=预测模型 | 30 |
| Cochrane library | #1(Risk prediction model):ti,ab,kw OR (Risk factor):ti,ab,kw OR (Predictor):ti,ab,kw OR (Model):ti,ab,kw OR (Risk Score):ti,ab,kw  #2(Enteral nutrition):ti,ab,kw OR (Feeding intolerance):ti,ab,kw OR (Feeding tolerance):ti,ab,kw  #3(Intensive care patient*):ti,ab,kw OR (Critically ill):ti,ab,kw OR (Critical illness*):ti,ab,kw OR (ICU*):ti,ab,kw  #4 #1AND#2AND#3 | 407 |
| pubmed | #1"enteral nutrition"[MeSH Terms] OR "feeding intolerance"[Title/Abstract] OR "feeding tolerance"[Title/Abstract]  #2"intensive care patient*"[Title/Abstract] OR "critically ill"[Title/Abstract] OR "critical illness*"[Title/Abstract] OR "ICU"[Title/Abstract]  #3"risk prediction model"[Title/Abstract] OR "risk factor"[Title/Abstract] OR "Predictor"[Title/Abstract] OR "Model"[Title/Abstract] OR "risk score"[Title/Abstract]  #1 AND #2 AND #3 | 169 |
| SinoMed | #1"喂养不耐受"[常用字段:智能] OR "肠内营养"[常用字段:智能]  #2"预测模型"[常用字段:智能]  #3 #1AND#2 | 28 |
| embase | #1'enteral nutrition':ab,ti OR 'feeding intolerance':ab,ti OR 'feeding tolerance':ab,ti  #2'intensive care patient*':ab,ti OR 'critically ill':ab,ti OR 'critical illness*':ab,ti OR icu*:ab,ti  #3'risk prediction model':ab,ti OR 'risk factor':ab,ti OR predictor:ab,ti OR model:ab,ti OR 'risk score':ab,ti  #4 #1AND#2AND#3 | 328 |
| CINAHL | S1 SU Enteral nutrition OR SU Feeding intolerance OR SU Feeding tolerance  S2 SU Intensive care patient* OR SU Critically ill OR SU Critical illness* OR SU ICU*  S3 SU Risk prediction model OR SU Risk factor OR SU Predictor OR SU Model OR SU Risk Score  S4 S1 AND S2 AND S3 | 188 |
| Web of Science | #1 Enteral nutrition (Title) or Feeding intolerance (Title) or Feeding tolerance (Title)  #2 Intensive care patient* (Title) or Critically ill (Title) or Critical illness* (Title) or ICU* (Title)  #3 Risk prediction model (Title) or Risk factor (Title) or Predictor (Title) or Model (Title) or Risk Score (Title)  #4 #1AND#2AND#3 | 32 |

# Supplementary Figures


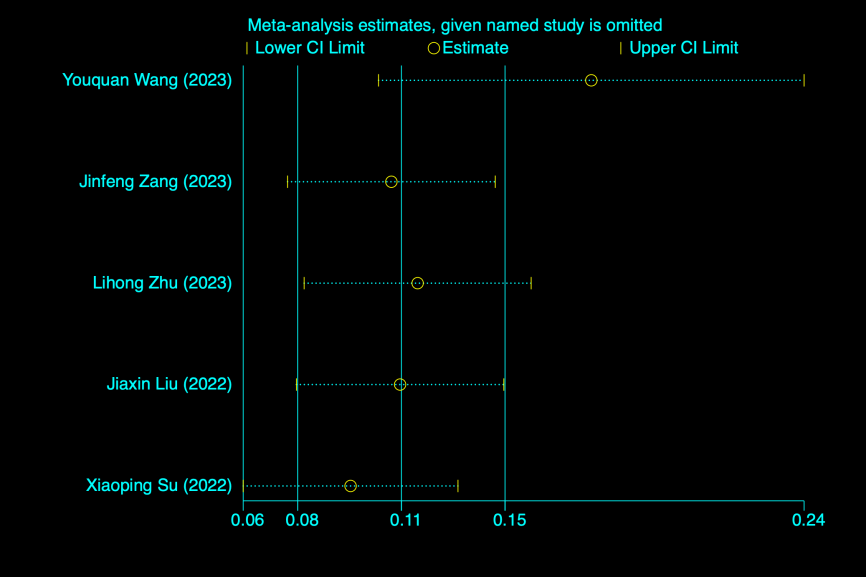


Supplementary Figure 1. Plot of sensitivity analysis of APACHE II score in predicting FI in patients with enteral nutrition.


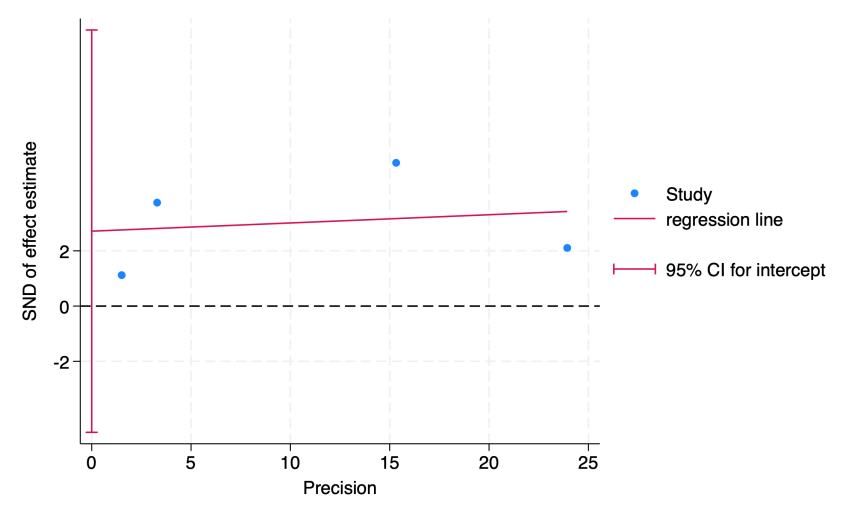


Supplementary Figure 2. Egger test for APACHE II score in predicting FI in patients with enteral nutrition.


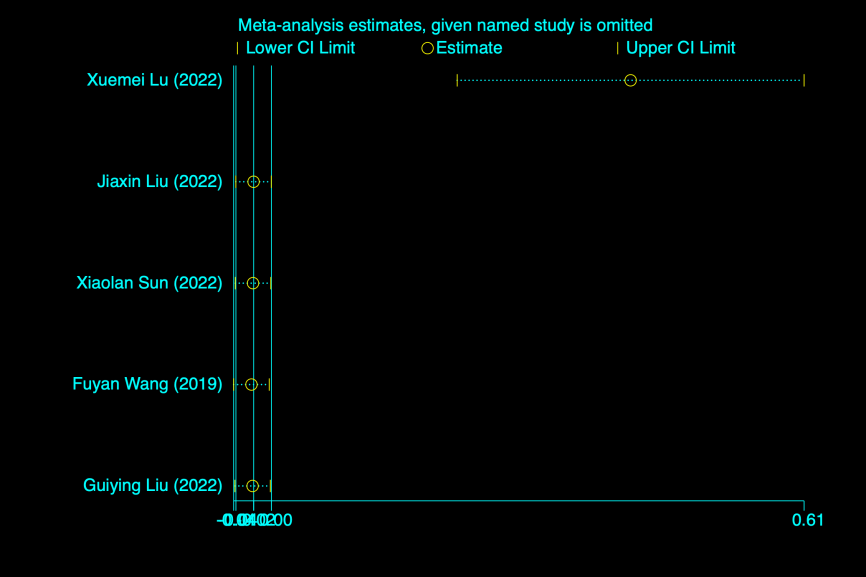


Supplementary Figure 3. Plot of sensitivity analysis of age in predicting FI in patients with enteral nutrition (1).


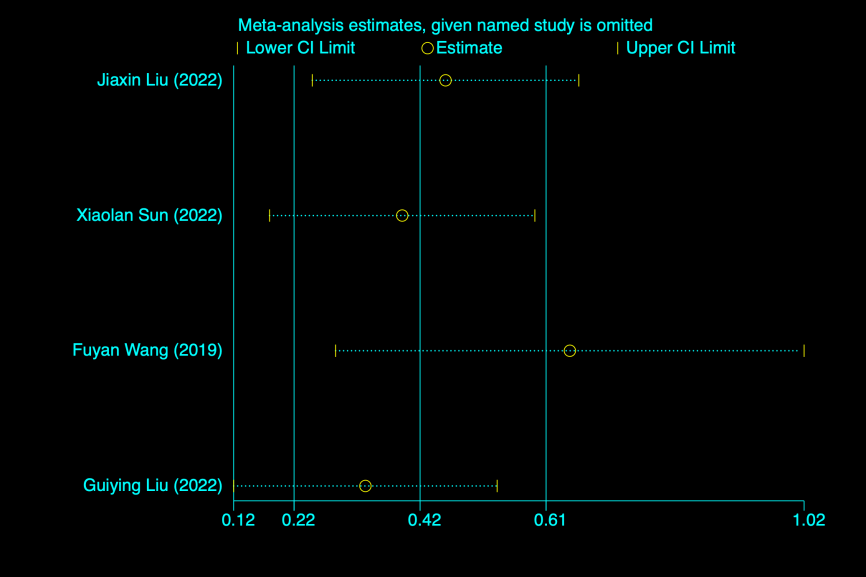


Supplementary Figure 4. Plot of sensitivity analysis of age in predicting FI in patients with enteral nutrition (2).


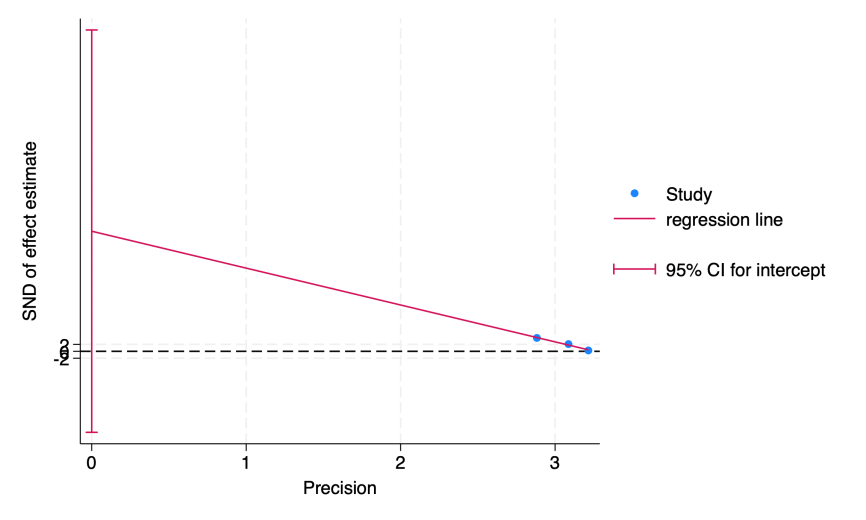


Supplementary Figure 5. Egger test for age in predicting FI in patients with enteral nutrition.


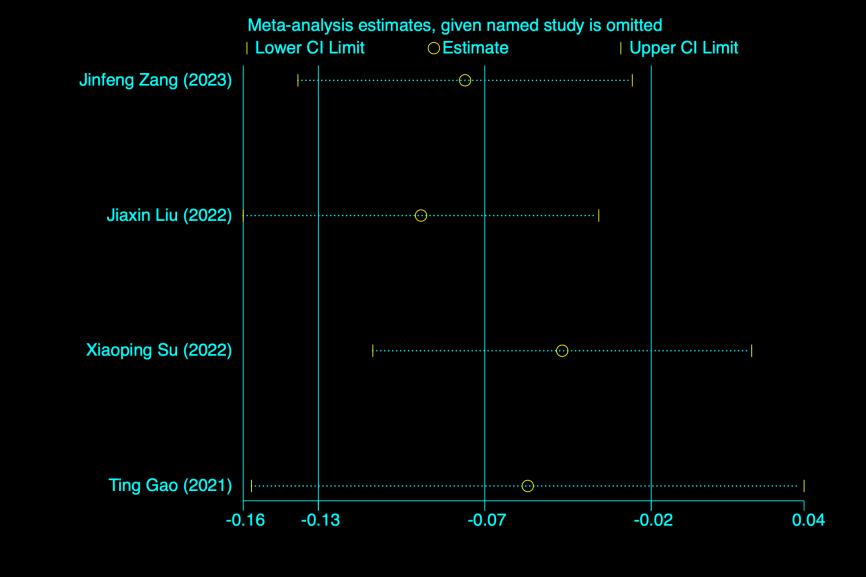


Supplementary Figure 6. Plot of sensitivity analysis of albumin levels in predicting FI in patients with enteral nutrition.


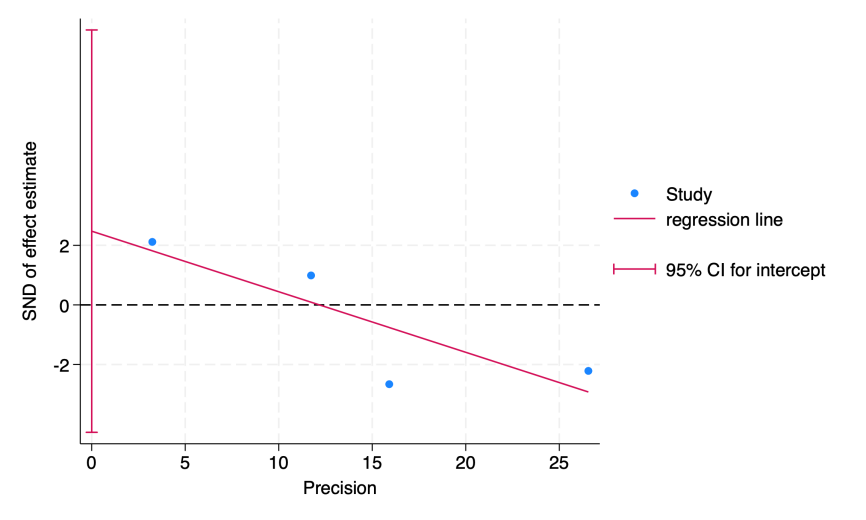


Supplementary Figure 7. Egger test for albumin levels in predicting FI in patients with enteral nutrition.


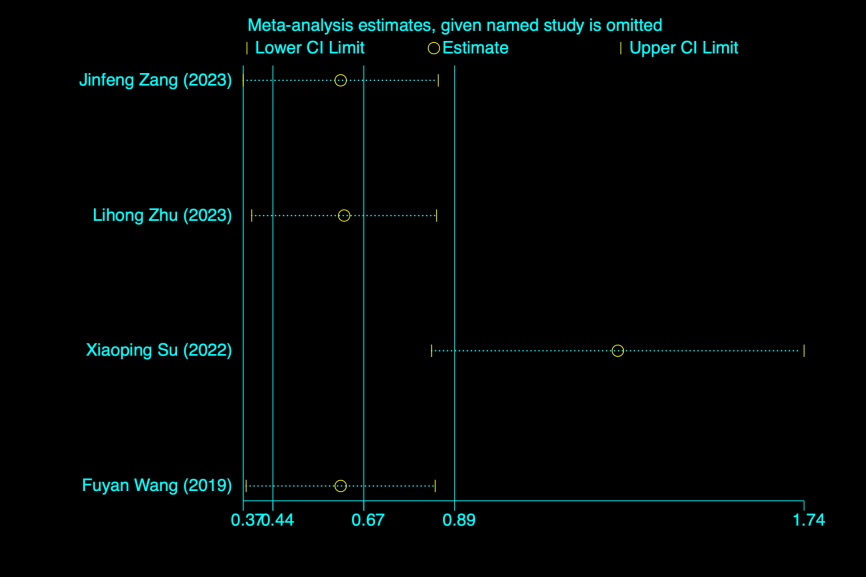


Supplementary Figure 8. Plot of sensitivity analysis of intra-abdominal pressure in predicting FI in patients with enteral nutrition.


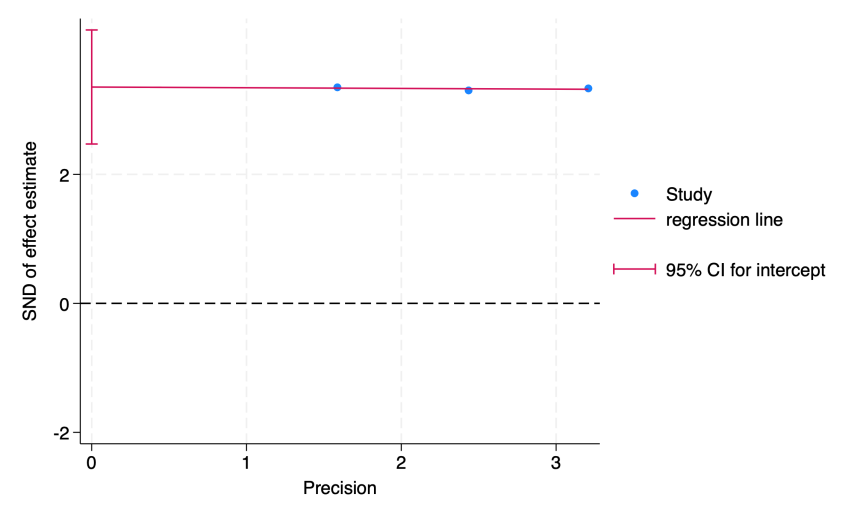


Supplementary Figure 9. Egger test for intra-abdominal pressure in predicting FI in patients with enteral nutrition.


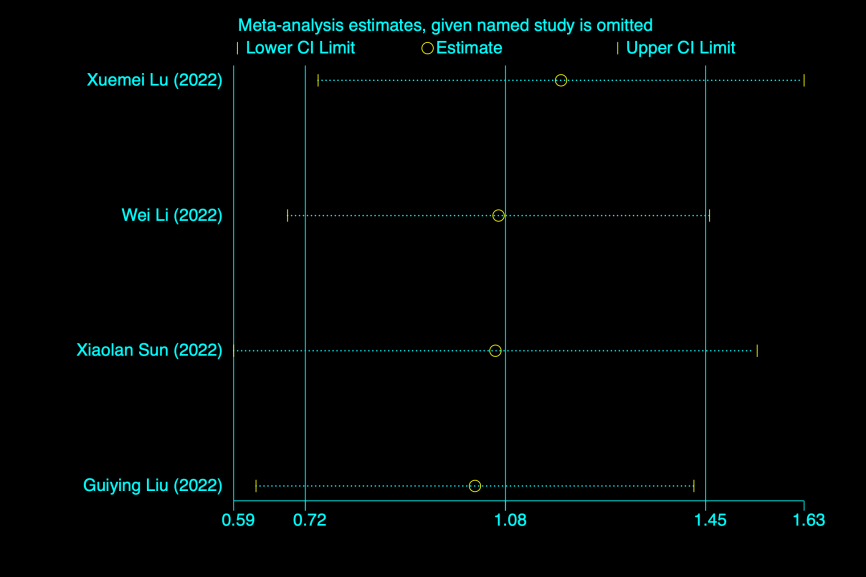


Supplementary Figure 10. Plot of sensitivity analysis of mechanical ventilation in predicting FI in patients with enteral nutrition.


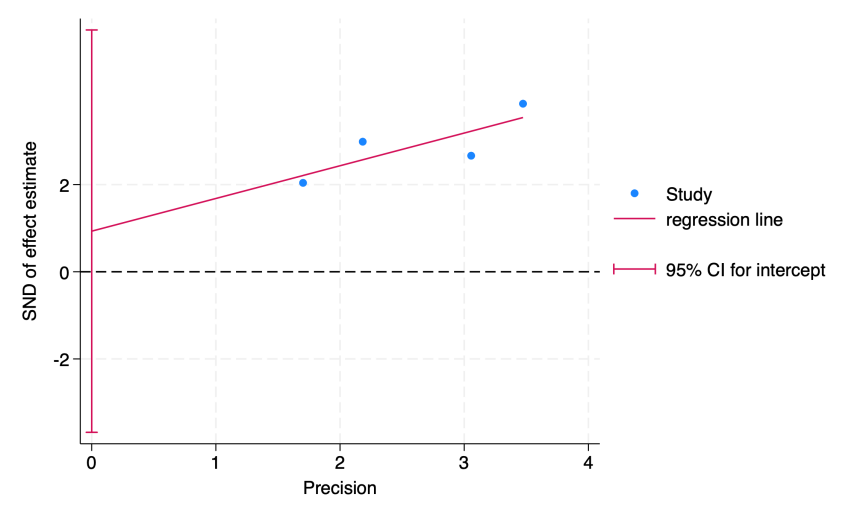


Supplementary Figure 11. Egger test for mechanical ventilation in predicting FI in patients with enteral nutrition.


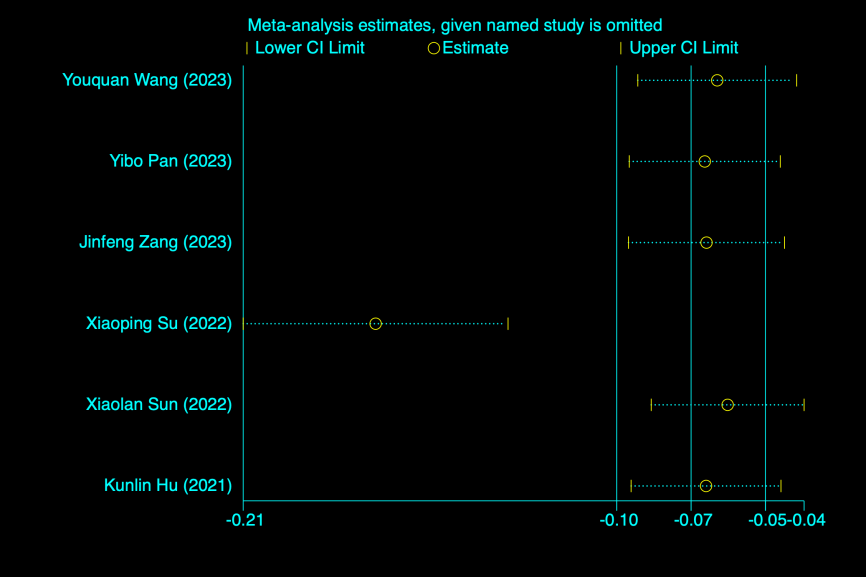


Supplementary Figure 12. Plot of sensitivity analysis of pooled AUC estimates for 6 validation models.


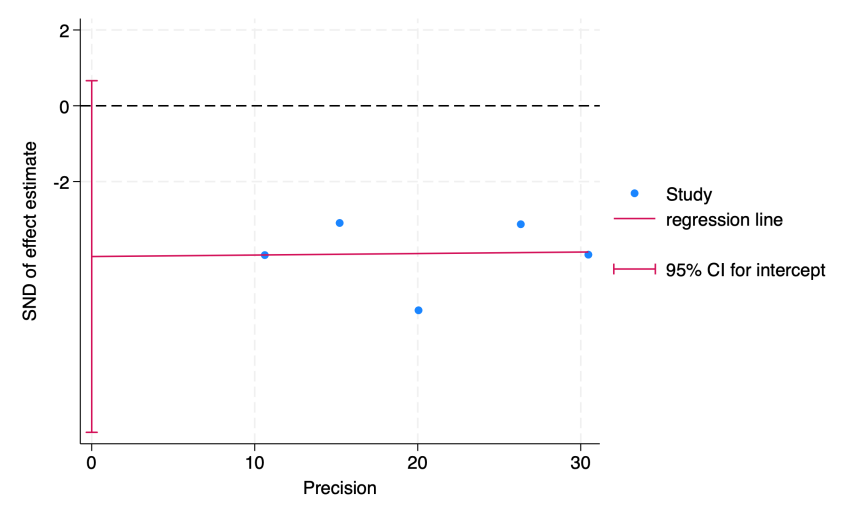


Supplementary Figure 13. Egger test for pooled AUC estimates for 5 validation models.
